# A Mindfulness-Based Resiliency Program for Caregivers of Patients With Severe Acute Brain Injury Transitioning Out of Critical Care: Protocol for an Open Pilot Trial

**DOI:** 10.2196/50860

**Published:** 2023-10-25

**Authors:** Alexander Mattia Presciutti, Emily Woodworth, Elizabeth Rochon, Molly Neale, Melissa Motta, Joseph Piazza, Ana-Maria Vranceanu, David Yi-Gin Hwang

**Affiliations:** 1 Department of Psychiatry Center for Health Outcomes and Interdisciplinary Research Massachusetts General Hospital Boston, MA United States; 2 Department of Psychiatry Harvard Medical School Boston, MA United States; 3 Department of Neurology Division of Neurocritical Care University of North Carolina School of Medicine Chapel Hill, NC United States; 4 Program in Trauma Department of Neurology University of Maryland School of Medicine Baltimore, MD United States

**Keywords:** severe acute brain injury, coma, caregiver, resilience, mindfulness, brain injury, emotional distress, decision-making, emotional support, intervention, support, self-report assessment, mental health

## Abstract

**Background:**

Caregivers of patients with severe acute brain injuries (SABI) that lead to coma and require intensive care unit (ICU) treatment often experience chronic emotional distress. To address this need, we developed the Coma Family (COMA-F) program, a mindfulness-based resiliency intervention for these caregivers.

**Objective:**

We will conduct an open pilot trial of COMA-F (National Institutes of Health Stage IA). Here we describe our study protocol and proposed intervention content.

**Methods:**

We will enroll 15 caregivers of patients with SABIs during their loved one’s hospital course from 3 enrollment centers. A clinical psychologist will deliver the COMA-F intervention (6 sessions) over Zoom (Zoom Video Communications, Inc) or in person. We will iterate COMA-F after each caregiver completes the intervention and an exit interview. English-speaking adults who have emotional distress confirmed by the clinical team and are the primary caregivers of a patient with SABI are eligible. The adult patient must have been admitted to the neuro-ICU for SABI and (1) have had a Glasgow Coma Scale score below 9 while not intubated or an inability to follow meaningful commands while intubated at any point during their hospitalization for >24 hours due to SABI; (2) will be undergoing either tracheostomy or percutaneous endoscopic or surgical gastrostomy tube placement or have already received one or both; and (3) have a prognosis of survival >3 months. We will identify eligible caregivers through screening patients’ medical records and through direct referrals from clinicians in the neuro-ICU. During the intervention we will teach caregivers mind-body and resilience skills, including deep breathing, mindfulness, meditation, dialectical thinking, acceptance, cognitive restructuring, effective communication, behavioral activation, and meaning-making*.* Caregivers will complete self-report assessments (measures of emotional distress and resilience) before and after the intervention. Primary outcomes are feasibility (recruitment, quantitative measures, adherence, and therapist fidelity) and acceptability (treatment satisfaction, credibility, and expectancy). We will conduct brief qualitative exit interviews to gather feedback on refining the program and study procedures. We will examine frequencies and proportions to determine feasibility and acceptability and will analyze qualitative exit interview data using thematic analysis. We will also conduct 2-tailed *t* tests to explore signals of improvement in emotional distress and treatment targets. We will then conduct an explanatory-sequential mixed methods analysis to integrate quantitative and qualitative data to refine the COMA-F manual and study procedures.

**Results:**

This study has been approved by the institutional review board at 1 of the 3 enrollment centers (2023P000536), with approvals at the other 2 centers pending. We anticipate that the study will be completed by late 2024.

**Conclusions:**

We will use our findings to refine the COMA-F intervention and prepare for a feasibility randomized controlled trial.

**Trial Registration:**

ClinicalTrials.gov NCT05761925; https://clinicaltrials.gov/study/NCT05761925

**International Registered Report Identifier (IRRID):**

PRR1-10.2196/50860

## Introduction

### Background

Severe acute brain injuries (SABI) such as ischemic stroke, hemorrhagic stroke, traumatic brain injury, or hypoxic-ischemic encephalopathy are sudden catastrophic events for both patients and their families [1]. The majority of patients with SABI cared for in an intensive care unit (ICU) are incapacitated, and many are comatose [2]. Caregivers of patients with SABI who survive their ICU admission nevertheless experience high rates of emotional distress, which, if untreated, becomes chronic [1,3]. These caregivers grapple with the uncertainty of their loved one’s long-term prognosis, the stress of making decisions on their loved one’s behalf, grief over their loved one’s loss of neurological function, and the stress of managing their own life outside of the ICU [1,3,4]. While the stay in an ICU itself can be quite lengthy, ultimate recovery from SABI can be prolonged and uncertain, and caregivers often endure these stressors beyond the acute hospitalization [5,6].

Caregivers of hospitalized patients with SABI have unmet needs for emotional support. Prior work shows that they feel overwhelmed, underprepared for caregiving, unable to manage basic self-care, isolated, and lonely, and that they deal with ambiguous loss [5-8]. Despite these needs, a recent systematic review found that among 22 interventions for caregivers to general ICU patients, none led to long-term improvements in emotional distress, and many actually increased caregiver burden [9]. Further, there are few interventions specific to the needs of SABI caregivers. Those that do exist focus mainly on crisis management and decision-making during the initial 48 hours of admission [3,10,11] rather than addressing prolonged uncertainty, grief, and stress. Taken together, there is a critical need for effective interventions for caregivers of surviving patients with SABI that extend into the period following the ICU stay.

**Figure 1:**
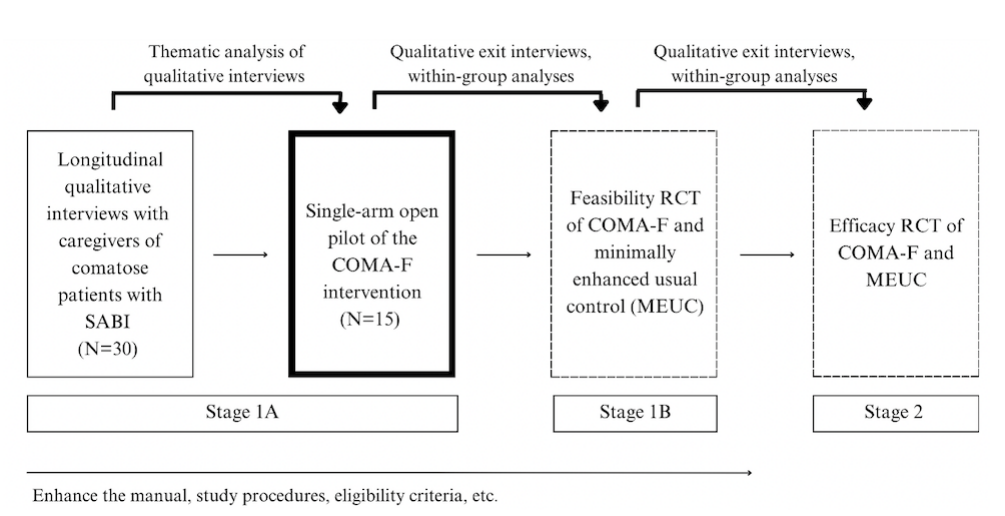
Iterative stages of the development of a mind-body resiliency program for caregivers of patients with severe acute brain injury (the COMA-F program). The open pilot trial described in this protocol is outlined in bold. The subsequent feasibility RCT and efficacy RCT are outlined by dashed lines. COMA-F: Coma Family program; MEUC: minimally enhanced usual control; RCT: randomized controlled trial; SABI: severe acute brain injury.

Our team has followed the National Institutes of Health (NIH) Stage Model for behavioral intervention development [12] to develop the Coma Family Program (COMA-F), a mindfulness-based resiliency program for caregivers of patients with SABI transitioning out of the ICU (Figure 1). We developed preliminary COMA-F content based on adaptations of our intervention for patients and caregivers hospitalized in the neuro-ICU (Recovering Together) [13] and from qualitative interviews of caregivers of surviving patients with SABI. For the latter, we conducted longitudinal qualitative interviews with 30 caregivers across 14 ICUs in the United States to better understand their stressors, coping methods, and treatment preferences [14,15]. Nearly all caregivers discussed using avoidance or distraction as initial coping strategies [14]. They also expressed a desire to learn adaptive coping skills, learn communication skills for interacting with the patient’s care team and other family members, and for support with making difficult decisions (Hwang et al, “Psychosocial support needs and preferences among family caregivers of ICU patients with severe acute brain injury: a qualitative thematic analysis,” manuscript under review). We have used this feedback and adapted content from Recovering Together to develop the first version of the COMA-F intervention. We plan to deliver COMA-F through a single-arm open pilot trial outlined in this paper (N=15 caregivers). We will use the findings from the open pilot to iteratively refine COMA-F. We will then conduct a feasibility randomized controlled trial (RCT) of COMA-F versus minimally enhanced usual control (MEUC).

### Objectives

Here we describe our procedure and delivery of the COMA-F open pilot trial. We will explore the preliminary feasibility and acceptability of COMA-F. Further, through exit interviews at the end of the intervention, we expect to elicit feedback on program procedures (hybrid in-person and Zoom format), treatment manuals, support from study staff, and expectations of the program. We will use this feedback to inform each iteration of COMA-F. After completion of the open pilot trial, we will then proceed to test the feasibility of the refined COMA-F program versus the MEUC.

## Methods

### Study Design and Setting

**Figure 2:**
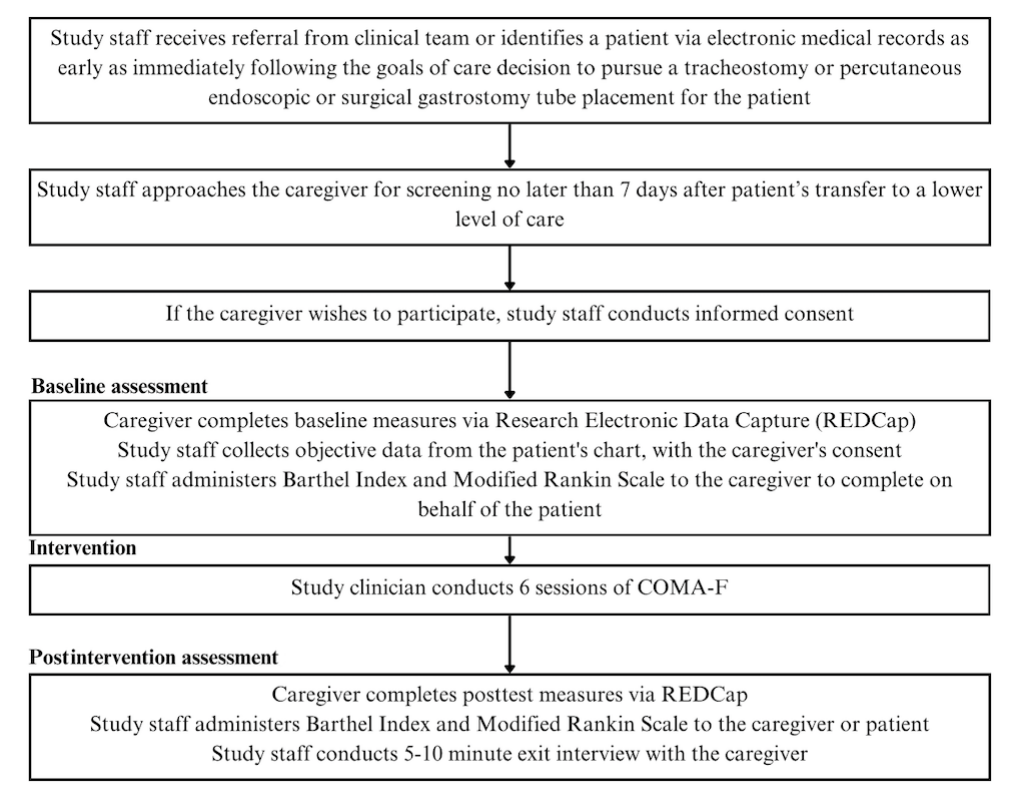
Open pilot trial design and procedure for the development of a mind-body resiliency program for caregivers of patients with severe acute brain injury (the COMA-F program). COMA-F: Coma Family; REDCap: Research Electronic Data Capture.

We will deliver our open pilot of COMA-F for caregivers of comatose patients with SABIs enrolled from 3 academic medical centers in the United States. If we encounter recruitment challenges, we will also plan to enroll from collaborators’ institutions in the northeastern and mid-Atlantic regions of the United States. We present our study design and procedure in Figure 2.

### Ethics Approval

This study has been approved by the institutional review board at 1 of the 3 enrollment centers (Mass General Brigham Institutional Review Board, 2023P000536), with approvals at the other 2 centers pending. We will conduct informed consent with all caregivers enrolled in the study. We will deidentify all data and provide subject ID numbers to participants. We will compensate participants US $20 via check for completing baseline assessments and US $40 via check for postintervention assessments, for a total of US $60 per participant.

### Recruitment and Screening

#### Overview

We present our study inclusion criteria in Textbox 1. We will recruit caregiver subjects through 2 concurrent mechanisms.

**Open pilot trial inclusion criteria for the development of a mind-body resiliency program for caregivers of patients with severe acute brain injury (the Coma Family program).**

- Age 18 years or older
- English-speaking
- Screens for emotional distress on either depression or anxiety subscales (>7) of the Hospital Anxiety and Depression Scale
- Confirmed by the primary clinical team as the primary caregiver for a patient who has the following characteristics:
  Age 18 years or older
  Admission to the intensive care unit with a severe acute brain injury for one of the following: ischemic stroke, intracerebral hemorrhage, subarachnoid hemorrhage, traumatic brain injury, or hypoxic-ischemic encephalopathy
  Glasgow Coma Scale score below 9 (in the judgment of the medical team) while not intubated or an inability to follow meaningful commands while intubated at any point during their hospitalization course for more than 24 consecutive hours due to the brain injury itself and not a confounding factor (ie, sedation and seizures)
  Still alive in the intensive care unit at the time that the clinical team approaches the primary caregiver about possible recruitment
  Either will be undergoing tracheostomy and percutaneous endoscopic or surgical gastrostomy tube placement or has already received one or both
  Has a prognosis for survival of greater than 3 months and does not have a concurrent diagnosis of a terminal illness or injury, as judged by the clinical team

#### Referrals From the Clinical Team

ICU medical and nursing team members who are caring for patients with SABIs will identify eligible caregivers and notify the study team, as they currently do in 1 of our enrollment centers for our ongoing Recovering Together trial of patients and caregivers in the neuro-ICU [13]. Notably, COMA-F will capture caregivers of patients with SABI as described in Textbox 1, whereas Recovering Together exclusively focuses on dyads of caregivers and patients who are cognitively intact.

#### Study Staff Screening

We will conduct daily prescreening of electronic health record admission reports to identify patients that meet the SABI criteria discussed below. Study staff will then inquire with the clinical team if the identified patients have a caregiver that meets inclusion criteria.

### Enrollment

Once a potential caregiver has been identified and approved by the clinical team, a member of the study team will approach the caregiver in person or call them via phone no later than 7 days after the patient’s transfer to a lower level of care.

Caregivers who wish to participate will complete consent. During the consent process we also inform caregivers that we will collect patient data from the medical record (eg, demographics, diagnosis, and procedures). Prior to the intervention, caregivers will complete self-report baseline assessments through Research Electronic Data Capture (REDCap) either in person on a tablet, or they will be emailed a link to the REDCap survey. We plan to enroll 15 caregivers.

### COMA-F Intervention Content

The intervention will have 6 sessions and be presented as a hybrid program. Caregivers will have the option for any number of initial sessions to be delivered in person within the hospital (until the patient is discharged). If the patient is discharged at any point in the intervention, the remaining sessions will be delivered via live video using Zoom. We will also give caregivers the option of completing the intervention completely over Zoom. Zoom is a free and secure internet-based videoconferencing software program that is currently used to provide care for patients at each of the recruiting centers.

Sessions focus on developing mindfulness-based resiliency skills to cope with and manage caregiver-related stressors. The sessions were developed based on caregiver feedback in our qualitative interviews [14,15] and from adapted Recovering Together content [13]. Caregivers will choose 6 modules from a selection of 7 modules. The 7 modules are presented in Table 1.

**Table 1:** Session outline for the COMA-F^a^ program. The program will be delivered to caregivers of patients with severe acute brain injuries. The program will take place over 6 weeks.

Session	COMA-F topic	COMA-F skills and session content
1	Coping with the here and now	Deep breathing, mindfulness, 24-hour block, and mindfulness meditation
2	Coping with uncertainty	Dialectics, hands as worries, and support with difficult decisions
3	Adjusting to new roles	Identify the distress spiral and challenge unhelpful thoughts
4	Engaging social support	Effective communication with other family members and friends and care team
5	Engaging with positive activities	Behavioral activation, using social support, and setting daily goals
6	Managing fear and worry	Observing fear and worry and acceptance and change
7	Making meaning from our experiences	Making meaning: finding purpose and meaning in new caregiving role

^a^COMA-F: Coma Family.

### Feasibility and Acceptability Outcomes

The feasibility and acceptability markers are presented in Table 2.

**Table 2:** Feasibility and acceptability outcomes for our open pilot trial of a mind-body resiliency program for caregivers of patients with severe acute brain injuries. Outcomes will be assessed at the end of the program.

Outcome		Definition
**Feasibility (>70% acceptable; >80% excellent)**		
	Recruitment	Percentage of eligible caregivers who participate
	Assessments	Percentage of enrolled caregivers with no measures missing
	Adherence	Percentage of caregivers that complete 4 of 6 sessions
	Therapist fidelity	After randomly selecting 20% of sessions, percentage of sessions with 100% of content delivered
**Acceptability (>70% acceptable; >80% excellent)**		
	Treatment satisfaction	Percentage of caregivers who score above the midpoint on the Client Satisfaction Questionnaire-3 [16]
	Credibility and expectancy	Percentage of caregivers who score above the midpoint on Credibility/Expectancy Questionnaire [17]
	Adverse events	Mild adverse events reported in <10% of caregivers; no adverse events linked to program participation

### Assessments

We selected quantitative measures based on those collected in Recovering Together and from our qualitative interviews during COMA-F development (Textbox 2). We will administer the same measures at baseline and after the intervention.

**Quantitative measures administer at baseline and after the intervention.**

**Emotional distress**

Hospital Anxiety and Depression Scale—measures anxiety and depression symptoms [18]; Posttraumatic Stress Disorder Checklist–5—measures posttraumatic stress symptoms [19].

**Resiliency factors**

Measure of Current Status Part A—measures adaptive coping abilities [20]; Cognitive and Affective Mindfulness Scale Revised—measures inclination toward acting mindfully [21]; Toronto Mindfulness Scale–Trait—measures trait mindfulness [22]; ENRICHD Social Support Instrument—measures perceived availability of social support [23].

**Functional status of patient**

Barthel Index—measures functional independence in activities of daily living [24]; Modified Rankin Scale—measures level of disability in patients after a SABI [25].

### Exit Interview Procedures

Caregivers will complete the postintervention measures listed above immediately after the end of session 6. We will then conduct a brief 5-10 minute exit interview. We will ask caregivers to provide feedback on the program procedures, hybrid in-person and Zoom format, treatment manuals, support from study staff, and expectations of the program. We will also ask caregivers to discuss their responses on the pre- and postintervention assessments.

### Data Analysis

For quantitative data, we will calculate frequencies and proportions of the feasibility and acceptability outcomes. We will also conduct exploratory analyses to examine differences in emotional distress outcomes and feasibility or acceptability outcomes by the sex of the caregiver. For qualitative data, we will examine exit interviews using thematic analysis following the Framework Method [26]. We will then conduct mixed methods analysis using an explanatory-sequential design which will allow us to integrate quantitative and qualitative data to refine the COMA-F manual and study procedures. Specifically, we will use the exit interviews to explain the quantitative findings and to elicit feedback on the program.

## Results

We have received funding by the Neurocritical Care Society to begin recruiting in January 2024. Based on our recruitment data from our qualitative interviews [14,15] we expect to enroll 15 caregivers by November 2024. Following this timeline, our last enrolled caregiver would complete the program in December 2024.

## Discussion

### Scientific Contribution

Chronic emotional distress is common in caregivers of comatose patients with SABIs, which in turn undermines their caregiving ability and the patient’s own health and recovery [27,28]. As novel neurocritical care interventions seek to pursue exciting and more aggressive measures to “cure coma,” [29-31] caregivers of more patients may potentially endure lengthy periods of uncertainty and distress. As such, the COMA-F program is timely, aiming to prevent the development of chronic distress in these caregivers by teaching them mindfulness-based resiliency skills. These skills will help caregivers manage the severity of their distress.

This protocol provides the framework of our open pilot trial of COMA-F. The intervention is novel and flexible, offering hybrid delivery methods to bypass geographical barriers to participation. Following the NIH Stage Model for behavioral intervention development [12], we will use the open pilot results to refine the program and inform a future feasibility RCT of COMA-F versus MEUC.

### Limitations

Our pilot trial has limitations that we hope to address in future iterations and adaptations of COMA-F. First, our recruitment will be restricted to the demographic distribution of our 3 enrolling centers. In our future feasibility RCT we will focus specifically on recruiting a representative racial and ethnic sample of the United States. Second, we are targeting caregivers caring for patients who are undergoing prolonged life-supporting therapy as opposed to transitioning patients to comfort care only. In future trials, we do plan to adapt COMA-F to be offered to caregivers potentially transitioning their loved ones to comfort measures.

### Conclusions

We will use the results from this open pilot trial to inform the development of a mind-body resiliency program for caregivers of patients with SABIs. Our study will provide quantitative and qualitative data that we will use to refine the current iteration of the program. We will then test the refined intervention in a feasibility RCT.

## Abbreviations

COMA-F: Coma Family
ICU: intensive care unit
MEUC: minimally enhanced usual control
NIH: National Institutes of Health
RCT: randomized controlled trial
REDCap: Research Electronic Data Capture
SABI: severe acute brain injury

## Data Availability

Data sharing is not applicable to this article as no data sets were generated or analyzed during this study.
